# Development and Clinical Validation of Model-Informed Precision Dosing for Everolimus in Liver Transplant Recipients

**DOI:** 10.1021/acsptsci.4c00581

**Published:** 2024-12-12

**Authors:** Jeayoon Lee, In-Wha Kim, Suk Kyun Hong, Nayoung Han, Kyung-Suk Suh, Jung Mi Oh

**Affiliations:** † College of Pharmacy and Research Institute of Pharmaceutical Sciences, Seoul National University, Seoul 08826, Republic of Korea; ‡ Department of Surgery, Seoul National University College of Medicine, Seoul 03080, Republic of Korea; § College of Pharmacy and Research Institute of Pharmaceutical Sciences, Jeju National University, Jeju, Special Self-Governing Province 63243, Republic of Korea; ∥ College of Pharmacy, Natural Products Research Institute, Seoul National University, Seoul 08826, Republic of Korea

**Keywords:** everolimus, population pharmacokinetics, liver transplantation, nonlinear mixed-effect modeling (NONMEM), model-informed precision dosing (MIPD)

## Abstract

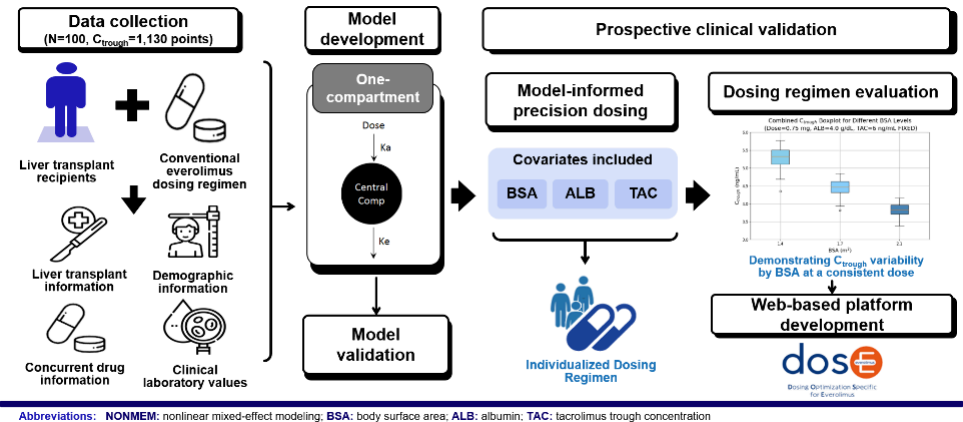

Everolimus presents significant dosing challenges due to between- and within-patient pharmacokinetic variabilities. This study aimed to develop and validate a model-informed precision dosing strategy for everolimus in liver transplant recipients. The dosing strategy was initially developed using retrospective data, employing nonlinear mixed-effects modeling. The model included readily measurable covariates, body surface area, albumin, and tacrolimus trough concentration. The dosing strategy was subsequently validated in a prospective trial, recommending 1 to 1.75 mg dosages every 12 h, depending on covariates. Lower dosages were recommended for patients with lower body surface area and albumin with adjustments based on tacrolimus trough concentration. The estimated pharmacokinetic parameters (typical value ± standard error), apparent clearance (CL/*F*: 15.0 ± 0.5 L/h), and apparent volume of distribution (V_d_/*F*: 862 ± 79.3 L) were refined using prospective clinical data from 20 patients, reducing interindividual variations. This research successfully developed and validated a population pharmacokinetic model for everolimus. The developed “dosE” web-based platform translates our pharmacokinetic model into a practical tool for healthcare providers, exemplifying the application of pharmaceutical research in clinical practice and potentially improving therapeutic outcomes in liver transplantation.

Everolimus, known for its anticancer and renal protective qualities, is a critical immunosuppressant in liver transplantation where individualized immunosuppressive therapy is crucial.^1−3^ While its therapeutic benefits in liver transplantation are notable, everolimus presents significant pharmacokinetic (PK) challenges, including substantial between- and within-patient variability. This variability is influenced by factors such as the P-glycoprotein transporter and metabolizing enzymes CYP3A4 and CYP3A5.^4^ Genetic variations in these enzymes and *ABCB1* can affect drug exposure and metabolism. These complexities are compounded in liver disease, where altered plasma protein levels and interactions with coadministered drugs can significantly impact drug metabolism and efficacy.^5^ Suboptimal dosing can lead to serious consequences, such as an increased risk of graft rejection and drug-induced toxicity, necessitating the development of a precisely tailored dosing strategy.

Everolimus needs therapeutic drug monitoring (TDM) to ensure optimal outcomes due to its narrow therapeutic index and significant interindividual exposure variability in exposure. When combined with other calcineurin inhibitors, everolimus dosage is generally adjusted to maintain trough concentrations (*C*_0_) within the 3–8 ng/mL range.^8^ Patients with blood concentrations below 3 ng/mL are at higher risk of acute rejection, potentially leading to graft loss, whereas those with blood concentrations above 8 ng/mL may experience a higher incidence of side effects such as thrombocytopenia and hypertriglyceridemia.^9−11^

However, relying solely on TDM may not always ensure therapeutic levels during critical periods and can be financially burdensome. This is particularly important in liver transplant recipients, where rapid improvements in liver function post-transplant necessitate tailored therapeutic approaches. In such cases, establishing a model-informed precision dosing (MIPD) strategy for everolimus becomes crucial for optimizing treatment outcomes. The cornerstone of MIPD is population pharmacokinetic (PopPK) modeling, which facilitates the transition from traditional one-size-fits-all treatments to personalized medicine by integrating individual patient data, drug concentrations, clinical profiles, and key physiological factors that influence PK.^12^

The need for MIPD is especially critical in the context of the high prevalence of living-donor liver transplantation. In these transplants, the limited size of the healthy donor liver graft may not meet the metabolic demands of the recipient, potentially leading to delayed liver function recovery and the life-threatening small-for-size graft syndrome.^13^ Furthermore, the smaller anatomical sizes of the liver graft’s hepatic veins, portal vein, arteries, and bile ducts complicate surgical techniques and increase the likelihood of postoperative complications.^14^

Previous PK studies have predominantly focused on renal and cardiac transplant recipients, highlighting the impact of factors such as ethnicity, liver function, and hematocrit levels on everolimus dosing.^6,9,10^ For example, Lemaitre et al. (2012) underscored the influence of liver function, demonstrating that increased bilirubin levels decrease everolimus clearance (CL), with variations in *CYP3A5* expression also contributing to CL differences, although not significantly.^7^ However, studies specifically tailored to liver transplant recipients are particularly scarce, and many existing models lack external validation. External validation trials are crucial for confirming the model’s accuracy and developing real-world dosing strategies. This study aims to develop and prospectively validate a MIPD strategy for everolimus to optimize its therapeutic efficacy and safety in liver transplant recipients.

## Results and Discussion

### Patient Characteristics in the PopPK Model

The characteristics of 100 patients for retrospective modeling are listed in Table 1. The mean age was 56 years, and 79% were male. Everolimus therapy was initiated at an average of 83 days after transplantation, with six different immunosuppressive regimens used, the most common being quadruple therapy, consisting of tacrolimus, mycophenolate mofetil, and prednisolone. Genotyping analysis revealed that all allelic frequencies were in Hardy–Weinberg equilibrium (*P* > 0.05), except for *CYP3A5* rs776746 (Supplementary Table 3). Consequently, *CYP3A5* was excluded from further analysis.

**Table 1:** Characteristics of 100 Korean Liver Transplant Recipients Taking Everolimus^a^

**Characteristics**	**Value**
**Age** (year)^b^	58 (22–75)
**Male**^c^	79 (79)
**Height** (cm)^d^	169.0 ± 6.3
**Weight** (kg)^d^	64.4 ± 8.8
**Body mass index** (kg/m^2^)^d^	22.5 ± 2.6
**Body surface area** (m^2^)^d^	1.7 ± 0.1
**Postoperative days** (day)^d^	82.8 ± 72.1
**Cause of transplantation**^c^	
Hepatitis B virus	53 (53)
Hepatitis C virus	7 (7)
Hepatitis B and C viruses	1 (1)
Alcohol-related liver disease	18 (18)
Alcohol-related liver disease, hepatitis B virus	6 (6)
Alcohol-related liver disease, hepatitis C virus	1 (1)
Others	14 (14)
**Presence of hepatocellular carcinoma**^c^	75 (75)
**Transplantation age** (year)^b^	57 (20–75)
**Model for end-stage liver disease score**^d^	12.93 ± 7.35
**Graft weight** (g)^d^	744.09 ± 243.15
**Donor male**^c^	56 (56)
**Donor age** (year)^b^	34 (16–72)
**Transplantation type**^c^	
Living	94 (94)
Deceased	6 (6)
**Human leukocyte antigen T-cell/B-cell match**^c^	
(−)/(−)	85 (85)
(−)/(+)	8 (8)
(+)/(+)	7 (7)
**ABO matched**^c^	
Matched	80 (80)
Unmatched	20 (20)
**Immunosuppressant agent regimen**^c^	
Everolimus/tacrolimus/mycophenolate mofetil/prednisolone	56 (56)
Everolimus/tacrolimus/mycophenolate mofetil	14 (14)
Everolimus/tacrolimus/prednisolone	18 (18)
Everolimus/mycophenolate mofetil/prednisolone	4 (4)
Everolimus/tacrolimus	6 (6)
Everolimus/prednisolone	2 (2)
**Laboratory data** (*N* = 85)^d^	
Hematocrit (%)	34.25 ± 5.69
Total protein (g/dL)	6.19 ± 0.75
Albumin (g/dL)	3.89 ± 0.50
Total bilirubin (mg/dL)	1.13 ± 1.22
Aspartate transaminase (IU/L)	38.42 ± 44.07
Alanine transaminase (IU/L)	68.92 ± 101.81
Serum creatinine (mg/dL)	1.33 ± 0.94
MDRD-GFR (mL/min/1.73 m^2^)	82.56 ± 38.75
hs-CRP (mg/L)	0.66 ± 2.10
**Tacrolimus trough concentration** (*N* = 79, ng/mL)^d^	8.29 ± 9.80

a Abbreviations: hs-CRP, high-sensitivity C-reactive protein; MDRD-GFR, modification of diet in renal disease equation glomerular filtration rate.

b Median (range).

c No. of patients (%).

d Mean ± SD.

### PopPK Model Development and Validation

A total of 1,130 everolimus whole blood concentrations were available for PopPK modeling. A one-compartment model with first-order absorption and elimination (ADVAN2 TRANS2) best-described everolimus PK. Interindividual variability was modeled exponentially, and a proportional error model accounted for the residual variability.

Correlation analyses revealed strong correlations between patient characteristics such as body weight and body mass index (BMI) (*r*^2^ = 0.763), and body weight and body surface area (BSA) (*r*^2^ = 0.974). Similarly, correlations were observed among laboratory parameters, namely, total protein with albumin (ALB) (*r*^2^ = 0.857) and aspartate transaminase with alanine transaminase (r^2^ = 0.865). Considering these strong correlations, only one of these covariates was included in the covariate analysis. ALB on volume of distribution (*V*_d_)/bioavailability (*F*) and ALB, BSA, and tacrolimus trough concentration (TAC) on *CL*/*F* exerted significant effects on the model during covariate model development using stepwise covariate modeling (SCM). All these covariates were statistically significant, with the impact of BSA on *CL*/*F* being clinically important based on the effect size estimate cutoff of 20%.^15^ None of the genotype covariates improved the model.

ALB, a key indicator of liver function, was identified as a crucial covariate, especially in patients with hepatic impairment—a frequent condition among liver transplant recipients. In these patients, ALB is linked to reduced CL of everolimus without altering protein binding.^16^ Contrary to common beliefs that lower ALB levels would increase *V*_d_ due to higher concentrations of free drugs, several studies have shown that free drug concentrations remain constant.^17,18^ Additionally, since everolimus extensively binds to erythrocytes—approximately 75% of its distribution within the erythrocytes—variations in ALB may influence erythrocyte deformability,^19^ thereby influencing the *V*_d_ of everolimus.

BSA, which incorporates both weight and height, serves as a more accurate reflection of the blood volume and correlates closely with the distribution of drugs such as everolimus within the vascular compartment.^20^ The well-documented correlation between BSA and everolimus CL influences dosing guidelines,^20^ particularly in oncology settings where BSA-based dosing adjustments are recommended.

Extensive research has been conducted on the interaction between TAC and everolimus.^21−24^ Both drugs are metabolized by the CYP3A enzyme system and are substrates for the P-glycoprotein transport system, leading to complex interactions that can affect drug levels. The competition at these key biological pathways can result in altered blood levels of everolimus, necessitating close monitoring and potentially requiring dose adjustments to maintain therapeutic effectiveness while avoiding toxicity. The interaction between tacrolimus and everolimus is particularly crucial due to their concurrent use in liver transplant recipients.

Potential influences of body weight, size, sex, age, and pharmacogenetic differences on everolimus levels were initially considered, but not selected as final covariates in our PopPK model, consistent with existing literature results.^6,7,25^ Due to the complex interplay of multiple genes in everolimus’ disposition, the individual genetic variations were not significant enough to be identified as key covariates. Furthermore, the impracticality of routine genetic testing in clinical settings limits the utility of genetic factors for daily management. In contrast, the covariates identified in our study (ALB, BSA, and TAC) are readily measurable. Therefore, they can be effectively utilized to optimize everolimus dosing, enhancing the practical application of our research findings in patient care.

Finally, the relationship between PK parameters and covariates is expressed in the following equations:
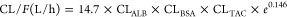

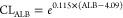

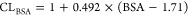

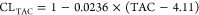

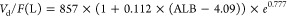

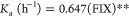
The final retrospective model’s predictive performance is presented in Table 2, with goodness of fit (GOF) plots shown in Supplementary Figure 1. The population means of *V*_d_/*F* and *CL*/*F* were 857 L and 14.7 L/h, respectively, with interindividual variabilities of 108.4% and 39.6%. Bootstrap and visual predictive check (VPC) data fit well within the fifth to 95th percentiles (Supplementary Figure 2).

**Table 2:** Population Pharmacokinetic Parameters of Everolimus in Liver Transplant Recipients^a^

					**Bootstrap**(*n* = 1000)	
**Pharmacokinetic parameter**	**Population mean**	**RSE (%)**	**Interindividual variability (%)**	**Shrinkage (%)**	**5th percentile**	**95th percentile**
**Fixed effects**						
*K*_a_ (1/h)	0.647 FIX	–	–	–	0.647	0.647
V_d_/*F* (L)	857.0	10.3	108.4	–	672.4	1090.5
CL/*F* (L/h)	14.7	4.1	39.6	–	13.8	15.8
CL ALB	0.115	5.6	–	–	0.063	0.165
CL BSA	0.492	101	–	–	–0.191	1.109
CL TAC	–0.0236	23.8	–	–	–0.0336	–0.0146
*V*_d_ ALB	0.112	14.4	–	–	0.078	0.140
**Random effects**						
ω^2^*V*_d_/*F*	0.777	15.6	–	27	0.491	1.040
ω^2^*CL*/*F*	0.146	14.6	–	3.9	0.114	0.184
Residual error (σ^2^), proportional	0.243	5.6	–	–	0.219	0.264

a Abbreviations: ALB, albumin; BSA, body surface area; CL, clearance; *F*, bioavailability; *K*_a_, absorption rate constant; RSE, relative standard error; TAC, tacrolimus trough concentration; *V*_d,_ volume of distribution; interindividual variability reported as % CV = sqrt(exp(OMEGA)–1) × 100; ω^2^, variance of the inter-individual random effects.

### Simulated Scenarios and Development of MIPD

**Figure 1:**
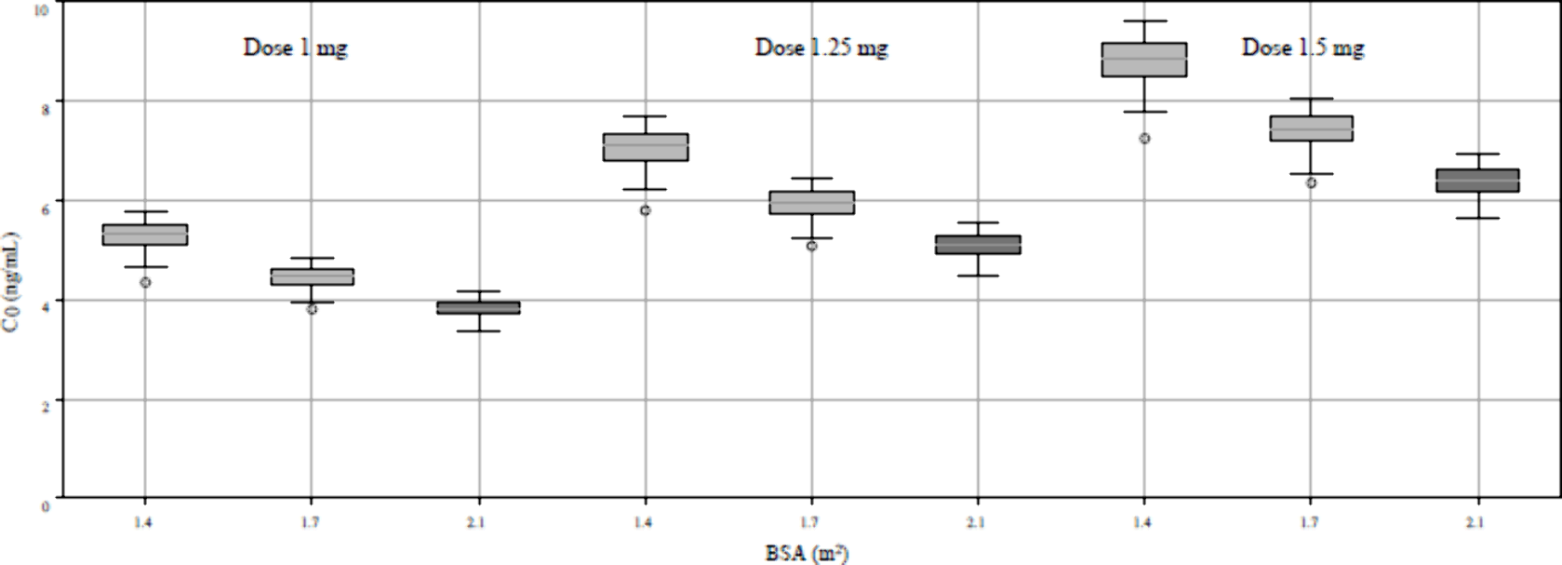
Combined C_trough_ boxplot for different dosing regimens and body surface areas.

To develop the 36 scenarios for evaluating the clinical utility of MIPD, representative values for each covariate were established. BSA was designated as low (1.4 m^2^), normal (1.7 m^2^), and high (2.1 m^2^). TAC was set at 6 and 10 ng/mL. Lastly, ALB was specified into low (2.5 g/dL) and normal (4.0 g/dL). Detailed descriptions are provided in Supplementary Table 4. Among the 36 simulated scenarios, the results for C_0_s were graphically represented by fixing the ALB at a normal value of 4.0 g/dL and the TAC at 6 ng/mL while varying the BSA and drug dosages (Figure 1).

Simulated data indicated that patients with higher BSA or ALB had lower everolimus whole blood levels, whereas others with higher TAC had higher everolimus whole blood levels with the same dosing. Based on these clinical covariates, the final MIPD was calculated for the prospective clinical trial, as shown in Table 3.

**Table 3:** Model-Informed Precision Dosage of Everolimus Based on Clinical Variables in Liver Transplant Recipients

**Clinical variables**			
**Albumin level (g/dL)**	**Tacrolimus trough concentration (ng/mL)**	**Body surface area (m**^**2**^**)**	**Model-informed precision dosage (mg)**
<3.5	<8	<1.6	1 mg every 12 h
		1.6–1.9	1.25 mg every 12 h
		>1.9	1.5 mg every 12 h
	≥8	<1.6	1 mg every 12 h
		1.6–1.9	1 mg every 12 h
		>1.9	1.25 mg every 12 h
≥3.5	<8	<1.6	1.25 mg every 12 h
		1.6–1.9	1.5 mg every 12 h
		>1.9	1.75 mg every 12 h
	≥8	<1.6	1 mg every 12 h
		1.6–1.9	1.25 mg every 12 h
		>1.9	1.5 mg every 12 h

### Patient Characteristics in the MIPD Prospective Trial

The characteristics of the 20 patients who received MIPD in the prospective clinical trial are provided in Table 4. All 20 participants underwent living-donor liver transplantation using two immunosuppressant regimens.

**Table 4:** Characteristics of the 20 Korean Liver Transplant Recipients Taking Model-Informed Precision Dosage of Everolimus^a^

**Characteristics**	**Value**
**Age** (year)^b^	58.5 (33–79)
**Male**^c^	16 (80)
**Height** (cm)^d^	166.24 ± 7.44
**Weight** (kg)^d^	67.83 ± 11.99
**Body mass index** (kg/m^2^)^d^	24.43 ± 3.42
**Body surface area** (m^2^)^d^	1.76 ± 0.19
**Postoperative days** (day)^d^	435.7 ± 697.99
**Cause of transplantation**^c^	
Hepatitis B virus	10 (50)
Hepatitis C virus	3 (15)
Alcohol-related liver disease	6 (30)
Alcohol-related liver disease, hepatitis B virus	1 (5)
**Presence of hepatocellular carcinoma**^c^	16 (80)
**Transplantation age** (year)^b^	58 (33–72)
**Model for end-stage liver disease score**^d^	16.72 ± 3.21
**Graft weight** (g)^d^	761.93 ± 128.64
**Donor male**^c^	11 (55)
**Donor age** (year)^b^	32.5 (20–56)
**Transplantation type**^c^	
Living	20 (100)
**Human leukocyte antigen T-cell/B-cell match**^c^	
(−)/(−)	18 (90)
(+)/(+)	2 (10)
**ABO matched**^c^	
Matched	17 (85)
Unmatched	3 (15)
**Immunosuppressant agent regimen**^c^	
Everolimus/tacrolimus/prednisolone	9 (45)
Everolimus/tacrolimus	11 (55)
**Laboratory data**^d^	
Hematocrit (%)	39.63 ± 4.48
Total protein (g/dL)	6.77 ± 0.52
Albumin (g/dL)	4.27 ± 0.36
Total bilirubin (mg/dL)	0.77 ± 0.31
Aspartate transaminase (IU/L)	19.35 ± 4.54
Alanine transaminase (IU/L)	22.4 ± 17.82
Serum creatinine (mg/dL)	0.92 ± 0.20
MDRD-GFR (mL/min/1.73 m^2^)	87.83 ± 16.69
hs-CRP (mg/L)	0.24 ± 0.28
**Tacrolimus trough concentration** (ng/mL)^d^	6.64 ± 1.88

a Abbreviations: hs-CRP, high-sensitivity C-reactive protein; MDRD-GFR, modification of diet in renal disease equation glomerular filtration rate.

b Median (range).

c No. of patients (%).

d Mean ± SD.

### Evaluation of the Predictive Power of MIPD

**Figure 2:**
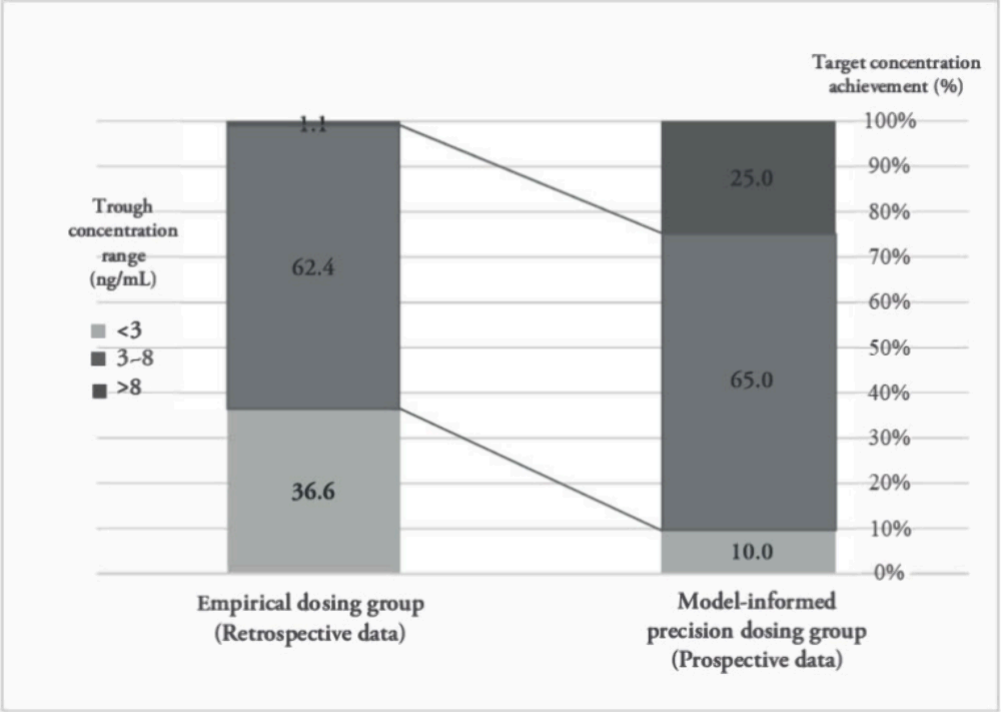
Achievement rates of everolimus target trough concentrations based on model-informed precision dosing.

The achievement rates of the target whole blood concentrations were assessed at the initial measurement for patients who received the MIPD (prospective data) and those who were given standard empirical dosages (retrospective data) (Figure 2). The target concentration achievement rate showed an upward trend in the MIPD group compared to the standard dosing group (65.0% vs 62.4%). While there was no statistical difference in the overall target achievement rates, the distribution within the target range improved notably with MIPD. There was an increase in overtarget concentrations and a decrease in undertarget concentrations, centering the distribution closer to the ideal target. Despite the observed increase in overtarget concentrations, during the 6-month follow-up period, no adverse events directly attributable to everolimus were reported. All adverse events occurred between 34 and 120 days post-initial dosing and were determined not to be causally related to the drug. This observation supports the safety of the MIPD strategy under current clinical monitoring protocols. However, continuous vigilance is advised to promptly address any potential toxicity due to elevated everolimus levels.

### Final Model Refinement

For model refinement, 79 whole blood drug concentrations were further collected from 20 patients who received the MIPD for a 6-month follow-up period. The incorporation of prospective patient data led to slight adjustments in PK parameters, as shown in Table 5 compared to the initial model in Table 2. Specifically, the population means for *V*_d_/*F* and *CL*/*F* showed minimal changes, with *V*_d_/*F* adjusting from 857.0 to 862.0 L and *CL*/*F* from 14.7 to 15.0 L/h, indicating a stable model structure and consistent parameter estimates. Importantly, these adjustments did not substantially alter the recommended dosage range of 1 to 1.75 mg every 12 h, due to the model’s inherent robustness.

**Table 5:** Final Population Pharmacokinetic Parameters of Everolimus in Liver Transplant Recipients^a^

					**Bootstrap**(*n* = 1000)	
**Pharmacokinetic parameter**	**Population mean**	**RSE (%)**	**Interindividual variability (%)**	**Shrinkage (%)**	**5th percentile**	**95th percentile**
**Fixed effects**						
*K*_a_ (1/h)	0.647 FIX	–	–	–	0.647	0.647
*V*_d_/*F* (L)	862.0	9.2	105	–	698.5	1082.0
CL/*F* (L/h)	15.0	3.3	36.8	–	14.2	15.9
CL ALB	0.122	22.9	–	–	0.072	0.165
CL BSA	0.474	91.4	–	–	–0.123	0.942
CL TAC	–0.026	17.9	–	–	–0.035	–0.018
*V*_d_ ALB	0.101	19.3	–	–	0.061	0.131
**Random effects**						
ω^2^*V*_d_/*F*	0.743	16.8	–	26.2	0.496	0.949
ω^2^*CL*/*F*	0.127	13.1	–	4.5	0.099	0.157
Residual error (σ^2^), proportional	0.242	5	–	–	0.221	0.263

a Abbreviations: ALB, albumin; BSA, body surface area; CL, clearance; *F*, bioavailability; *K*_a_, absorption rate constant; RSE, relative standard error; TAC, tacrolimus trough concentration; *V*_d_, volume of distribution; interindividual variability reported as % CV = sqrt(exp(OMEGA)–1) × 100); ω^2^, variance of the inter-individual random effects.

The refined model incorporated the same covariates (ALB, BSA, and TAC), while the additional data improved precision in interindividual variability estimates for *CL*/*F* and *V*_d_/*F*. The variability in *CL*/*F* decreased from 39.6% to 36.8% and in *V*_d_/*F* from 108.4% to 105%. This reduction in variability enhances dose predictability and allows for more precise adjustments based on individual patient covariates, contributing to a more reliable MIPD approach. The narrower distribution around the target concentration range improves the therapeutic consistency across diverse patient profiles.

**Figure 3:**
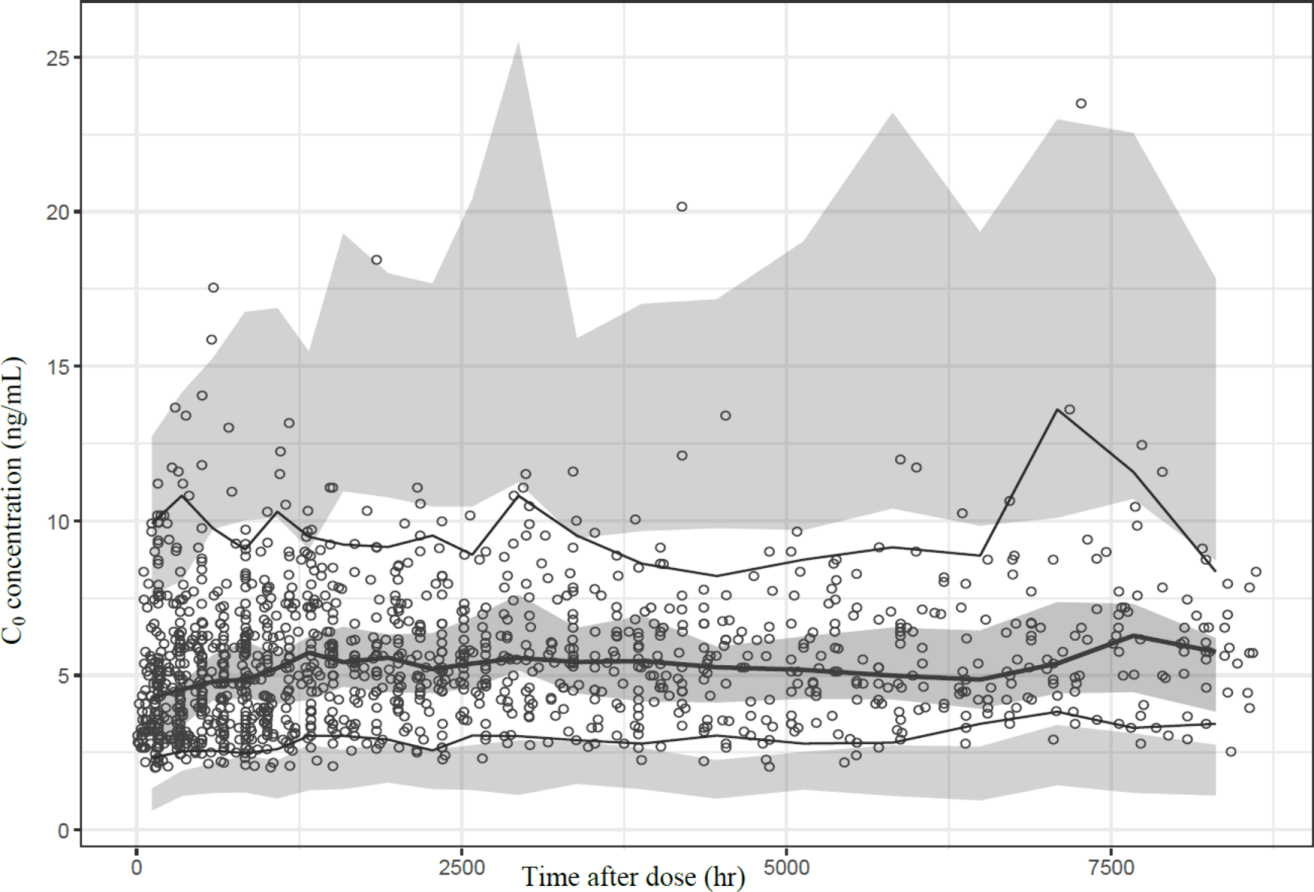
Visual predictive check for the final refined population pharmacokinetic model of everolimus in liver transplant recipients. The semibold lines represent the fifth and 95th percentiles of the prediction-corrected concentrations. The bold line represents the 50th percentile of the prediction-corrected concentrations. The semitransparent field represents a simulation-based 95% confidence interval.

As shown in Table 5, the data for everolimus fit well within the fifth to 95th percentiles of the bootstrap results. The GOF plots of the final model are presented in Supplementary Figure 3. The refined model’s predictive performance was enhanced, with narrower percentile ranges in the VPC (Figure 3). Based on the refined model, the web-based platform “dosE” was developed to assist healthcare providers in optimizing everolimus dosing.

Our PopPK model, refined with both retrospective and prospective data, was most accurately represented by a one-compartment model with first-order absorption and elimination. This selection aligns with the PK profile commonly observed in clinical practice where only *C*_0_s are routinely measured.^6,26^ The *CL*/*F* and *V*_d_/*F* estimates generated from our model closely match those derived from our retrospective data and values found in established literature,^27−29^ reinforcing their validity.

This study prospectively validated the MIPD of everolimus in liver transplant recipients, introducing a novel dosage adjustment method that utilizes readily measurable clinical factors, including those that can be assessed even in outpatient visits. This innovation allows for more precise initial dosing and provides a customized, patient-specific regimen, supported by the improved achievement rates of everolimus compared to those of conventional methods. Although the difference may appear modest, the distribution effectively centered closer to the ideal target. This shift demonstrates a clinically meaningful refinement of dose accuracy, highlighting the effectiveness of the MIPD strategy. Moreover, the refined model slightly reduced interindividual variations, which indicated enhanced predictability of the everolimus level and improved patient outcomes.

This improved target achievement rate is particularly crucial in the context of liver transplantation, where achieving and maintaining optimal drug levels can significantly impact patient outcomes. The development of the web-based platform “dosE” demonstrates the direct clinical application of our study, providing valuable information for clinicians to tailor everolimus dosing strategies. Clinicians seeking to utilize “dosE” can access it at https://dose.snu.ac.kr/ by requesting an access code from the corresponding author (JO).

The study’s prospective validation of the MIPD strategy, as applied in a clinical trial setting, represents a significant step forward in external validation for everolimus dosing in liver transplant recipients. Unlike previous studies that focused largely on renal and cardiac transplantation without subsequent validation, our approach integrated real-world clinical application and specifically targeted the existing gap in liver transplant PK research. By using a trial-based validation, we demonstrated a practical application of MIPD that adjusts to the dynamic conditions of liver transplantation, enhancing dosing accuracy and therapeutic outcomes.

Although this study has made significant strides, it also has several limitations. First, the initial model was built by using retrospective data, which may contain discrepancies between recorded and actual drug administration times. Second, the patient cohort consisted exclusively of Korean patients, characterized by lower BMI, a higher incidence of hepatocellular carcinoma, and a notable proportion of living donor-living transplantation. All of these demographic characteristics were thoroughly tested and considered in the covariate analysis. This approach ensured that the model could be adjusted to enhance its applicability across diverse populations, even though not all tested characteristics were selected as final covariates. Despite these challenges, the derived insights were pivotal for the reinforcement and validation of the retrospective model, significantly improving its practical application in clinical settings. This underscores the model’s reliability and safety for clinical application, affirming its value in improving patient outcomes.

In conclusion, this study highlights the crucial role of PopPK modeling in refining everolimus therapy for liver transplant recipients. By integrating key covariates such as ALB, BSA, and TAC, the MIPD strategy has effectively improved target achievement rates and reduced variability among individuals. The prospective validation of the model confirmed its efficacy, with a significantly higher proportion of patients achieving the desired therapeutic range of 3–8 ng/mL. Additionally, the development of the “dosE” platform, which is directly informed by these findings, represents a major advancement toward personalized immunosuppressive therapy in liver transplantation. Therefore, this research successfully demonstrates the potential of the MIPD strategy to enhance treatment outcomes for liver transplant recipients, underscoring its value in clinical practice.

## Methods

### Patients and Data Collection for PopPK Model

#### Eligibility

This retrospective study was conducted at a tertiary hospital in Korea, involving liver transplant recipients who received everolimus (Certican; Novartis Pharma AG, Basel, Switzerland) treatment from June 2015 to February 2021. Eligibility criteria included participants who had available data on everolimus trough concentrations and who had consented to the use of their biological samples for genetic analysis. Non-Korean individuals were excluded. The study was approved by the Institutional Review Board of Seoul National University Hospital (IRB No. 2105-125-1220), in accordance with the Declaration of Helsinki, and did not require additional patient consent due to its retrospective nature.

Patients who were prescribed everolimus twice daily combined with tacrolimus, mycophenolate mofetil, and steroids, as part of their immunosuppressive regimen, were included in this study. Initial dosages of everolimus ranged from 0.25 to 2.5 mg, determined by clinical judgment, and were subsequently adjusted based on TDM results to maintain the target C_0_ within the range of 3–8 ng/mL. Blood samples were collected retrospectively from outpatient visits over a one-year period, and everolimus whole blood concentrations were quantified using liquid chromatography with tandem mass spectrometry (LC–MS/MS) technique.

#### Genotyping Analysis

Genomic DNA was extracted from blood samples using the QuickGene DNA Whole Blood Kit S (Kurabo, Osaka, Japan) following the manufacturer’s instructions. For genotypic analysis, the SNaPshot assay targeted *CYP3A5 (6986A > G)*, *ABCB1 (3435C > T*, *2677T > G/A)*, and *PIK3R1 (3025A > G)*. The Taqman assay was employed for *POR* (1508C > T), *NR1I2* (7635G > A, 25385C > T), and *FGFR4* (1162G > A). These genes were selected based on PharmGKB data, which indicate genetic variations that have been observed to contribute to PK variability in the metabolism of everolimus and sirolimus. Details on SNaPShot and Taqman assay methods are provided in Supporting Information (Supplementary Tables 1 and 2). Adherence to the Hardy–Weinberg equilibrium was assessed using the χ^2^ test.

#### Retrospective PopPK Modeling and Validation

The PopPK model was established by the following steps: (1) construction of the most appropriate structural model with residual errors using both fixed and random effects, (2) investigation of potential covariates to explain the PK variability, and (3) internal validation of the developed model.

PopPK modeling was performed using the nonlinear mixed-effect modeling (NONMEM) program version 7.5.0 (ICON Development Solutions, Dublin, Ireland) and Pirana version 2.9.7 (Certara, Princeton, NJ, USA). Xpose 4 graphical diagnostics in R software version 4.1.0 (The R Foundation for Statistical Computing, Vienna, Austria) was used to evaluate the GOF.

#### Structural Model

One- or two-compartment PK models with or without lag time were evaluated. Drug disposition was compared using a combination of zero- or first-order absorption with linear or nonlinear elimination. For the first-order absorption model, the absorption rate constant was fixed at a value reported previously,^29^ as only *C*_0_s were available, with no data from the absorption phase to consider. Because everolimus’ *F* could not be identified, the values of CL and *V*_d_ were equal to the *CL*/*F* and *V*_d_/*F* ratios. Both proportional and exponential models were used to estimate the interindividual variabilities of PK parameters. Additive, proportional, and a combination of both models were tested for residual errors. Moreover, we explored incorporating the omega matrix to better model the variability between CL and Vd. The selection of the best model was based on the lowest Akaike information criterion.

#### Covariate Model

A comprehensive list of covariates was examined to elucidate PK variability including demographic, clinical, and genetic factors. These encompassed sex, age, height, body weight, BMI, BSA, postoperative days, transplantation type, age at transplantation, model for end-stage liver disease score, cause of transplantation (previous diagnosis), donor sex, donor age, graft weight, clinical laboratory values (such as hematocrit, total protein, ALB, total bilirubin, liver function values, and renal function values), genotypes, dose of the coadministered immunosuppressants (tacrolimus, mycophenolate mofetil, and steroids), and TAC.

Initially, the covariate correlation was tested, and only one from each group of covariates with strong associations (correlation coefficients > 0.7) was selected for inclusion in the candidate models. Then, a SCM method was used to determine the relationships between parameters and covariates for a systematic covariate search. Categorical covariates were estimated using the additive models, whereas continuous covariates used additive, exponential, and power models. The continuous covariates were normalized to the population median values. The heterogeneity of all genotypes was tested under the dominant, recessive, and additive models. For the forward inclusion of SCM, the statistical significance level was set to *P* < 0.05, which corresponded to a 3.84 change in the objective function value (OFV). For backward elimination of SCM, the significance level was set to *P* < 0.01, which corresponded to at least a 6.63 change in the OFV, with both for one degree of freedom. All NONMEM analyses were performed using the first-order conditional estimation method with the interaction option.

#### Model Validation

The resampling techniques of bootstrap (*n* = 1000) and VPC (*n* = 1000) were performed to evaluate the accuracy and robustness of the final model. The medians and 5th and 95th percentiles of the bootstrap result parameters were compared with the values from the original data. Bootstrap and VPC were performed with Perl-speaks-NONMEM (PsN) version 5.3.1 (Uppsala University, Uppsala, Sweden).

### Prospective Validation of Retrospective PopPK Model

#### Simulation-Based Dosing Optimization

To develop the dosing nomogram, scenarios were created for a hypothetical population, and random data were generated from the model’s probability distribution. For every 50 participants, whole blood concentration–time profiles were simulated using NONMEM after giving three doses of 1.0, 1.25 and 1.5 mg of everolimus.

#### Study Design

A prospective, nonrandomized, single-arm trial was conducted to evaluate the effectiveness of the MIPD strategy in adult liver transplant recipients initiating everolimus therapy. Before enrollment, all participants were fully informed about the study’s purpose, procedures, and alternative treatment options, and provided written informed consent. The study protocol was approved by the Ethics Committee and Institutional Review Board of Seoul National University Hospital (IRB No. 2112–162–1287) and was conducted in accordance with the Declaration of Helsinki.

#### Participants

The included participants were adults aged ≥18 years, initiating everolimus therapy as part of their immunosuppressive regimen after liver transplantation. Patients were excluded if they underwent simultaneous organ transplantations or were taking drugs known to affect the PK of everolimus including antifungal agents (itraconazole, ketoconazole, posaconazole, and voriconazole), antibiotics (clarithromycin, telithromycin, and rifampin), or verapamil. Patients with a history of everolimus hypersensitivity, those in a medical crisis, and any individuals deemed by the researchers as inappropriate to fulfill the study requirements were also excluded.

#### MIPD Strategy

Everolimus was administered based on MIPD that considered three clinical variables, namely, ALB, BSA, and TAC. The drug was administered twice daily every 12 h. The specific dose was calculated considering each patient’s *CL*/*F* and *V*_d_/*F* (equation provided below), which is used to describe the *C*_0_ at a steady state of multiple oral doses.^30^
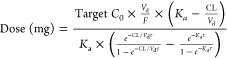


#### Outcomes

The primary outcome was the achievement rate (%), defined as the percentage of patients achieving the target *C*_0_ range on day 7 following the initiation of everolimus therapy. We compared the achievement rates between patients treated with MIPD and those receiving conventional empirical dosages. The χ2 test was employed to assess differences in the achievement of therapeutic targets between MIPD and conventional dosing groups. Achievement rates were calculated as percentages for each group, and the statistical significance of the differences was evaluated. Statistical analyses were conducted using IBM SPSS Statistics version 20 (IBM Corp., Armonk, NY, USA) with a *p*-value less than 0.05 was considered statistically significant.

### Final Model Refining

This research involved improving the PopPK model of everolimus by integrating whole blood concentration levels from both retrospective and prospective data. Blood sampling was performed precisely before the morning dose on outpatient visit days, initiated on day 7, and continued for 6 months after everolimus initiation. The whole blood concentrations of everolimus were analyzed by using an electrochemiluminescence immunoassay. Originally, the retrospective model used data acquired by LC–MS/MS; however, additional data were gathered utilizing immunoassays. To reconcile these disparities, concentrations acquired from the immunoassay were adjusted using current literature data to align with previous concentration values.^31^

The model was refined following the same procedures as for the retrospective PopPK modeling. The refined final PopPK model validation was performed by using a GOF plot and VPC methods. GOF plots were used to assess the agreement between observed and predicted values, while VPC was employed to evaluate the model’s ability to predict the distribution of real-world data. Through these validation processes, the predictive performance and clinical applicability of the refined model were evaluated. Leveraging the insights gained from this refined model, we developed “dosE”, a web-based dosing platform, an acronym for “dosing optimization specific for everolimus.”

## Supporting Information Available

Additional experimental details, materials, and methods, including genotype data; Supplementary Figure 1 Goodness of fit plots obtained from the retrospective model; Supplementary Figure 2: VPC for the retrospective population PK model of everolimus in liver transplant recipients; Supplementary Figure 3: Goodness of fit plots obtained from the final model (PDF)

## Abbreviations

ALB: albumin
BMI: body mass index
BSA: body surface area
*C*_0_: trough concentration
CL: clearance
*F*: bioavailability
GOF: goodness of fit
hs-CRP: high sensitivity C-reactive protein
*K*_a_: absorption rate constant
LC-MS/MS: liquid chromatography- with tandem mass spectrometry
MDRD-GFR: modification of diet in renal disease equation glomerular filtration rate
MIPD: model-informed precision dosing
NONMEM: nonlinear mixed-effect modeling
OFV: objective function value
PK: pharmacokinetic
popPK: population pharmacokinetic
RSE: relative standard error
SCM: stepwise covariate modeling
TAC: tacrolimus trough concentration
TDM: therapeutic drug monitoring
*V*_d_: volume of distribution
VPC: visual predictive check
ω^2^: variance of interindividual random effects
