# Supplementary material for: Development and Clinical Validation of Model-Informed Precision Dosing for Everolimus in Liver Transplant Recipients
Source: ACS Pharmacol Transl Sci. 2024 Dec 12;8(1):216–24. doi: 10.1021/acsptsci.4c00581 (PMC11729436; doi:10.1021/acsptsci.4c00581)

Supporting information

Development and clinical validation of model-informed precision dosing for everolimus in liver transplant recipients

Jeayoon Lee[a], In-Wha Kim[a], Suk Kyun Hong[b] , Nayoung Han[c], Kyung-Suk Suh[b], Jung Mi Oh[a,d*]

[a]College of Pharmacy and Research Institute of Pharmaceutical Sciences, Seoul National University, Seoul, Republic of Korea.

[b]Department of Surgery, Seoul National University College of Medicine, Seoul, Republic of Korea.

[c]College of Pharmacy and Research Institute of Pharmaceutical Sciences, Jeju National University, Jeju Special Self-Governing Province, Republic of Korea.

[d]College of Pharmacy, Natural Products Research Institute, Seoul National University, Seoul, Republic of Korea.

[*]E-mail: jmoh@snu.ac.kr

Corresponding Author

Jung Mi Oh

College of Pharmacy and Research Institute of Pharmaceutical Sciences, Natural Products Research Institute,

Seoul National University, 1 Gwanak-ro, Gwanak-gu, Seoul 08826, Korea

Tel: 82-2-880-7997, Fax: 82-2-882-9560

ORCID No.: 0000-0002-1836-1707; E-mail: jmoh@snu.ac.kr

Contents

Supplementary Material 1 DNA direct sequencing, SNaPshot assay, and Taqman assay

Table S1 Primer sets and melting temperature (Tm) for the SNaPshot assay

Table S2 Probe type and assay ID for the Taqman assay

Table S3 Genotypes of the 100 Korean liver transplant recipients taking everolimus

Table S4 Simulation scenarios for everolimus dosages in liver transplant recipients

Figure S1 Goodness of fit plots obtained from the retrospective model

Figure S2 VPC for the retrospective population PK model of everolimus in liver transplant recipients

Figure S3 Goodness of fit plots obtained from the final model

Supplementary Material 1 DNA direct sequencing, SNaPshot assay, and Taqman assay

SNaPShot assay

The genotyping of *PIK3R1* (rs10515074), *CYP3A5* (rs776746), and *ABCB1* (rs1045642 and rs2032582) polymorphisms was screened utilizing a single-base primer extension assay using an ABI PRISM SNaPShot Multiplex kit (Applied Biosystems, Foster City, CA, USA) according to the manufacturer's recommendation. Briefly, the genomic DNA flanking the interested single-nucleotide polymorphism was amplified with polymerase chain reactions (PCRs) with forward and reverse primer pairs (Supplementary Table 1). The PCRs were performed as follows: 10 min at 95°C for 1 cycle, 35 cycles at 95°C for 30 s, 60°C for 1 min, and 72°C for 1 min, followed by 1 cycle at 72°C for 10 min. After amplification, the PCR products were purified. Then, 1 μL of the purified amplification products was added to a SNaPshot Multiplex Ready reaction mixture containing 0.15 pmol of the genotyping primer for the primer extension reaction. The primer extension reaction was performed for 25 cycles of 96°C for 10 s, 50°C for 5 s, and 60°C for 30 s. The reaction products were analyzed by electrophoresis in an ABI Prism 3730xl DNA analyzer (Applied Biosystems). Analysis was performed using GeneMapper software 4.0 (Applied Biosystems).

TaqMan assay

The genotyping of *POR* (rs1057868), *FGFR4* (rs1057868), and *NR1I2* (rs6785049 and rs3814055) polymorphisms was screened using the TaqMan Fluorogenic 5' Nuclease Assay (Applied Biosystems) with Assay Mix (Assay ID: rs2032582; C8890131_30, rs351855; C3166614_10, rs6785049; rs6785049, rs3814055; C__27504984_30) (Supplementary Table 2). The thermal cycle conditions were as follows: 95°C for 15 min to activate the DNA polymerase, followed by 45 cycles of 95°C for 15 s and 60°C for 1 min. All PCRs were performed using 384-well plates by a Dual 384-Well GeneAmp PCR System 9700 (Applied Biosystems). The endpoint fluorescent readings were performed on an ABI PRISM 7900 HT Sequence Detection System (Applied Biosystems). To ensure the accuracy of the genotyping, duplicate samples and negative controls were included.

Table S1 Primer sets and melting temperature (Tm) for the SNaPshot assay

Gene	rs number	Strand	Primer sequence		Tm	Additive
PIK3R1	rs10515074	Forward	Forward primer	TTTTCAGAAACTTGGTCCAACA		
			Reverse primer	TGCAACTCTCATGCAACCA	60	-
			Genotyping primer	GCATTTGCCAGCCCARCAAT		
CYP3A5	rs776746	Forward	Forward primer	TTATGGAGAGTGGCATAGGA		
			Reverse primer	GCTGATTAAACTTCACTAGCC	60	-
			Genotyping	CTCTTTAAAGAGMTCTTTTGTCTTTCA		
ABCB1	rs1045642	Forward	Forward primer	tcttgttttcagCTGCTTG	55	Betaine
			Reverse primer	aacccaaacaggaagtgtg		
			Forward primer	GGTGGTGWCACAGGAAGAGAT		
			Reverse primer	CAAATCTTGGGACAGGAAT		
			Genotyping primer	ggcctgaaaactgaaaaag		
ABCB1	rs2032582	Forward	Reverse primer	TGGAATTTGTCCCAGAGCA	55	Betaine
			Genotyping primer	tgttttctttgcttagag		
			Reverse primer	AATTCCCAGAGCTAAAGCA		

Table S2 Probe type and assay ID for the Taqman assay

Gene	rs number	Concentration	Assay ID
POR	rs1057868	40×	C8890131_30
FGFR4	rs351855	40×	C3166614_10
NR1I2	rs6785049	40×	C29280426_10
NR1I2	rs3814055	40×	C27504984_30

Table S3 Genotypes of the 100 Korean liver transplant recipients taking everolimus

Gene	Variant	Genotype	Frequency (%)	Minor allele frequency	*P*-value
CYP3A5	6986A>G	AA	8.67	A (0.299)	0.01*
	(rs776746)	AG/GA	40.66		
		GG	47.67		
ABCB1	3435C>T	CC	40.69	T (0.349)	0.56
	(rs1045642)	CT/TC	43.62		
		TT	11.69		
	2677T>G/A	TT	11.23	A (0.196)	0.83
	(rs2032582)	TG/GT	30.62	T (0.340)	
		TA/AT	12.93		
		AG/GA	17.63		
		GG	20.88		
		AA	3.72		
POR	1508C>T	CC	31.51	T (0.427)	0.53
	(rs1057868)	CT/TC	46.98		
		TT	17.51		
NR1I2	7635G>A	GG	26.29	A (0.479)	0.60
	(rs6785049)	GA/AG	28.42		
		AA	22.29		
	25385C>T	CC	54.75	T (0.245)	0.33
	(rs3814055)	CT/TC	35.49		
		TT	5.75		
FGFR4	1162G>A	GG	33.25	A (0.411)	0.92
	(rs351855)	GA/AG	46.49		
		AA	16.25		
PIK3R1	3025A>G	AA	58.59	G (0.219)	0.40
	(rs10515074)	AG/GA	32.81		
		GG	4.59		

* *CYP3A5* deviated from the Hardy–Weinberg equilibrium

Table S4 Simulation scenarios for everolimus dosages in liver transplant recipients

Scenario #	Dose (mg)	Body surface area (m^2)	Albumin (g/dL)	Tacrolimus level (ng/mL)
1	1	Low (1.4)	Low (2.5)	6
2	1	Normal (1.75)	Low (2.5)	6
3	1	High (2.1)	Low (2.5)	6
4	1	Low (1.4)	Normal (4.0)	6
5	1	Normal (1.75)	Normal (4.0)	6
6	1	High (2.1)	Normal (4.0)	6
7	1	Low (1.4)	Low (2.5)	10
8	1	Normal (1.75)	Low (2.5)	10
9	1	High (2.1)	Low (2.5)	10
10	1	Low (1.4)	Normal (4.0)	10
11	1	Normal (1.75)	Normal (4.0)	10
12	1	High (2.1)	Normal (4.0)	10
13	1.25	Low (1.4)	Low (2.5)	6
14	1.25	Normal (1.75)	Low (2.5)	6
15	1.25	High (2.1)	Low (2.5)	6
16	1.25	Low (1.4)	Normal (4.0)	6
17	1.25	Normal (1.75)	Normal (4.0)	6
18	1.25	High (2.1)	Normal (4.0)	6
19	1.25	Low (1.4)	Low (2.5)	10
20	1.25	Normal (1.75)	Low (2.5)	10
21	1.25	High (2.1)	Low (2.5)	10
22	1.25	Low (1.4)	Normal (4.0)	10
23	1.25	Normal (1.75)	Normal (4.0)	10
24	1.25	High (2.1)	Normal (4.0)	10
25	1.5	Low (1.4)	Low (2.5)	6
26	1.5	Normal (1.75)	Low (2.5)	6
27	1.5	High (2.1)	Low (2.5)	6

28	1.5	Low (1.4)	Normal (4.0)	6
29	1.5	Normal (1.75)	Normal (4.0)	6
30	1.5	High (2.1)	Normal (4.0)	6
31	1.5	Low (1.4)	Low (2.5)	10
32	1.5	Normal (1.75)	Low (2.5)	10
33	1.5	High (2.1)	Low (2.5)	10
34	1.5	Low (1.4)	Normal (4.0)	10
35	1.5	Normal (1.75)	Normal (4.0)	10
36	1.5	High (2.1)	Normal (4.0)	10

Figure S1 Goodness of fit plots obtained from the retrospective model

(a) Individually predicted everolimus concentrations (IPRED) (ng/mL) versus observed everolimus concentrations (dependent variable [DV]) (ng/mL),

(b) Population predicted everolimus concentration (PRED) (ng/mL) versus observed everolimus concentrations (DV) (ng/mL),

(c) Conditional weighted residuals (CWRES) versus population predicted everolimus concentration (PRED) (ng/mL),

(d) Conditional weighted residuals (CWRES) versus time of observation (TIME) (hours), where time 0 is the first administration time of everolimus

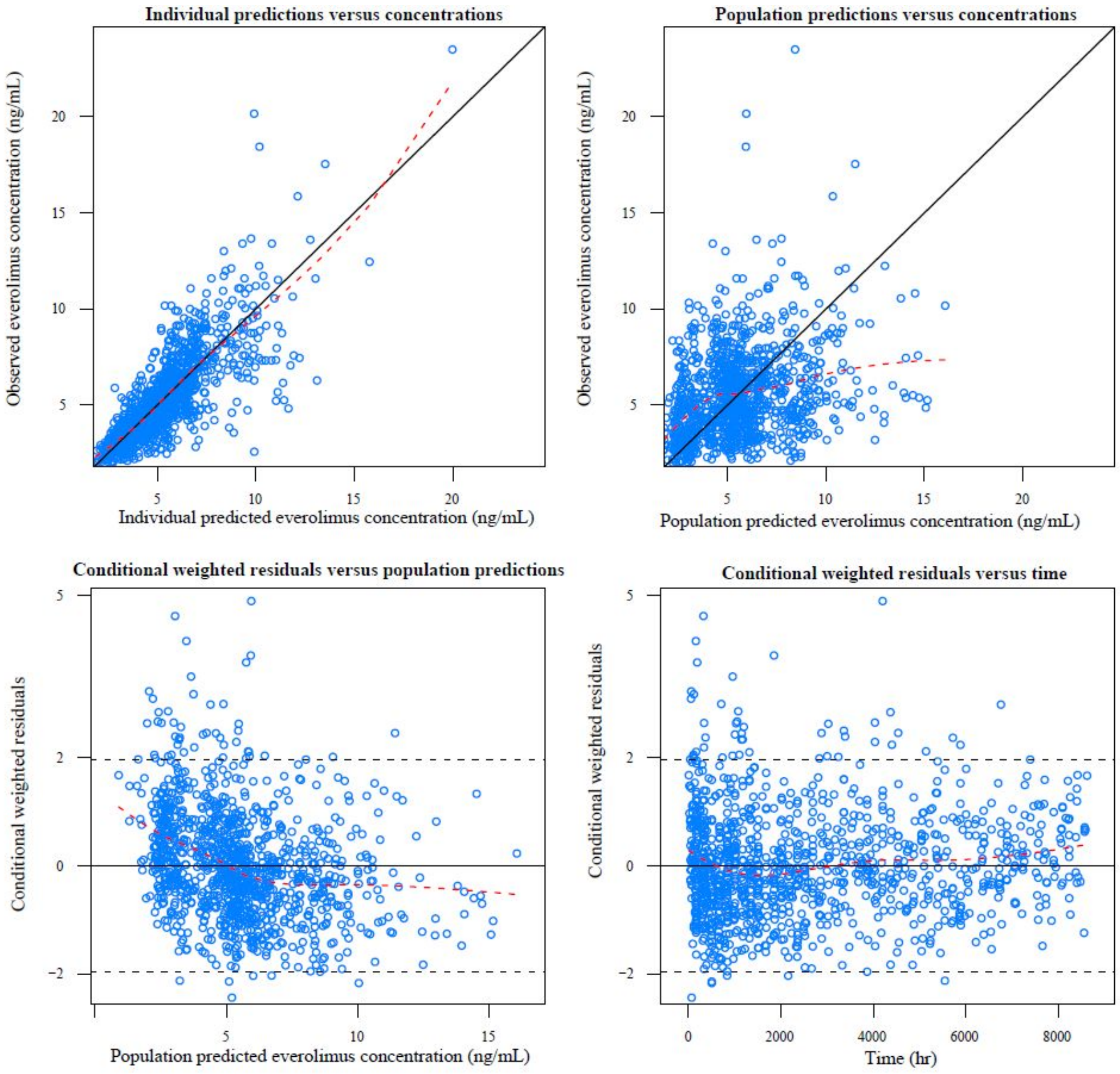

Figure S2 VPC for the retrospective population PK model of everolimus in liver transplant recipients

The semi-bold lines represent the 5th and 95th percentiles of the prediction-corrected concentrations. The bold line represents the 50th percentile of the prediction-corrected concentrations. The semitransparent field represents a simulation-based 95% confidence interval.

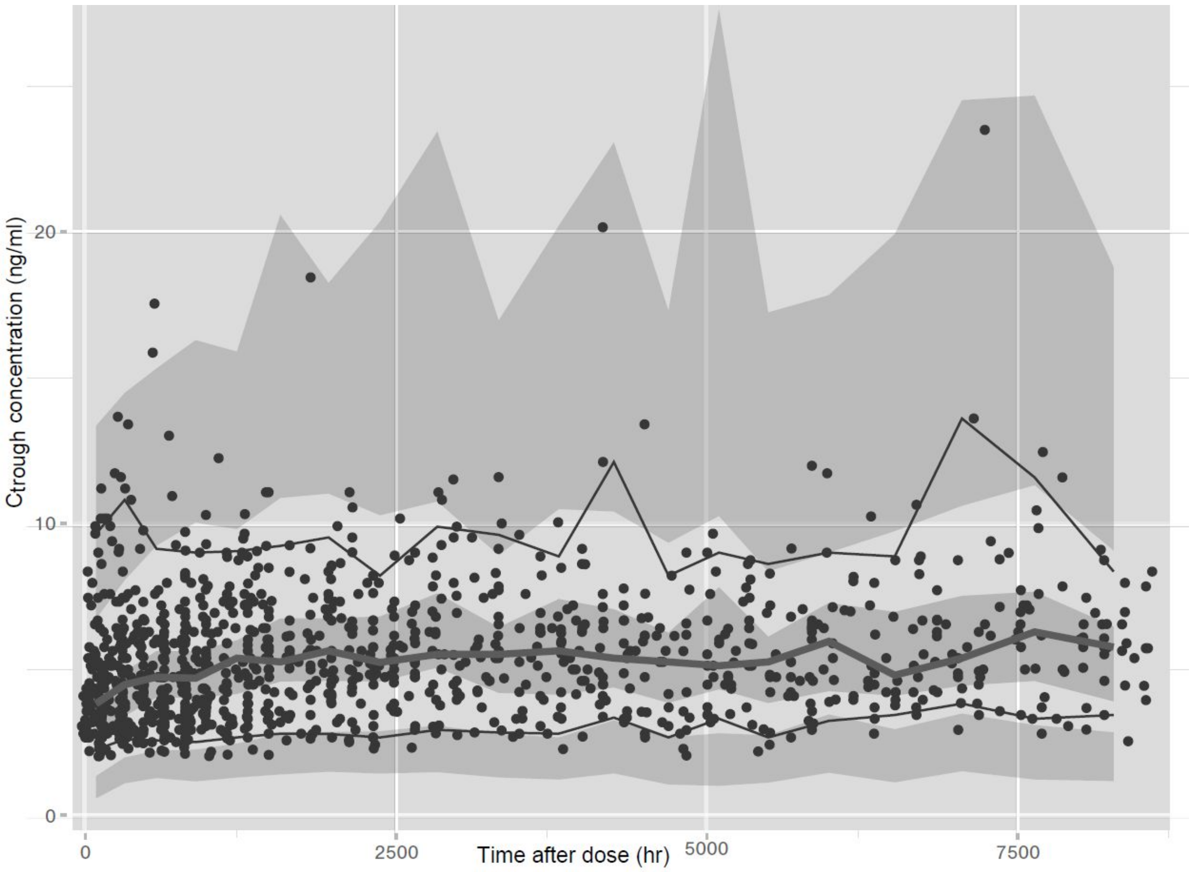

Figure S3 Goodness of fit plots obtained from the final model

(a) Individually predicted everolimus concentrations (IPRED) (ng/mL) versus observed everolimus concentrations (dependent variable [DV]) (ng/mL),

(b) Population predicted everolimus concentration (PRED) (ng/mL) versus observed everolimus concentrations (DV) (ng/mL),

(c) Conditional weighted residuals (CWRES) versus population predicted everolimus concentration (PRED) (ng/mL),

(d) Conditional weighted residuals (CWRES) versus time of observation (TIME) (hours), where time 0 is the first administration time of everolimus

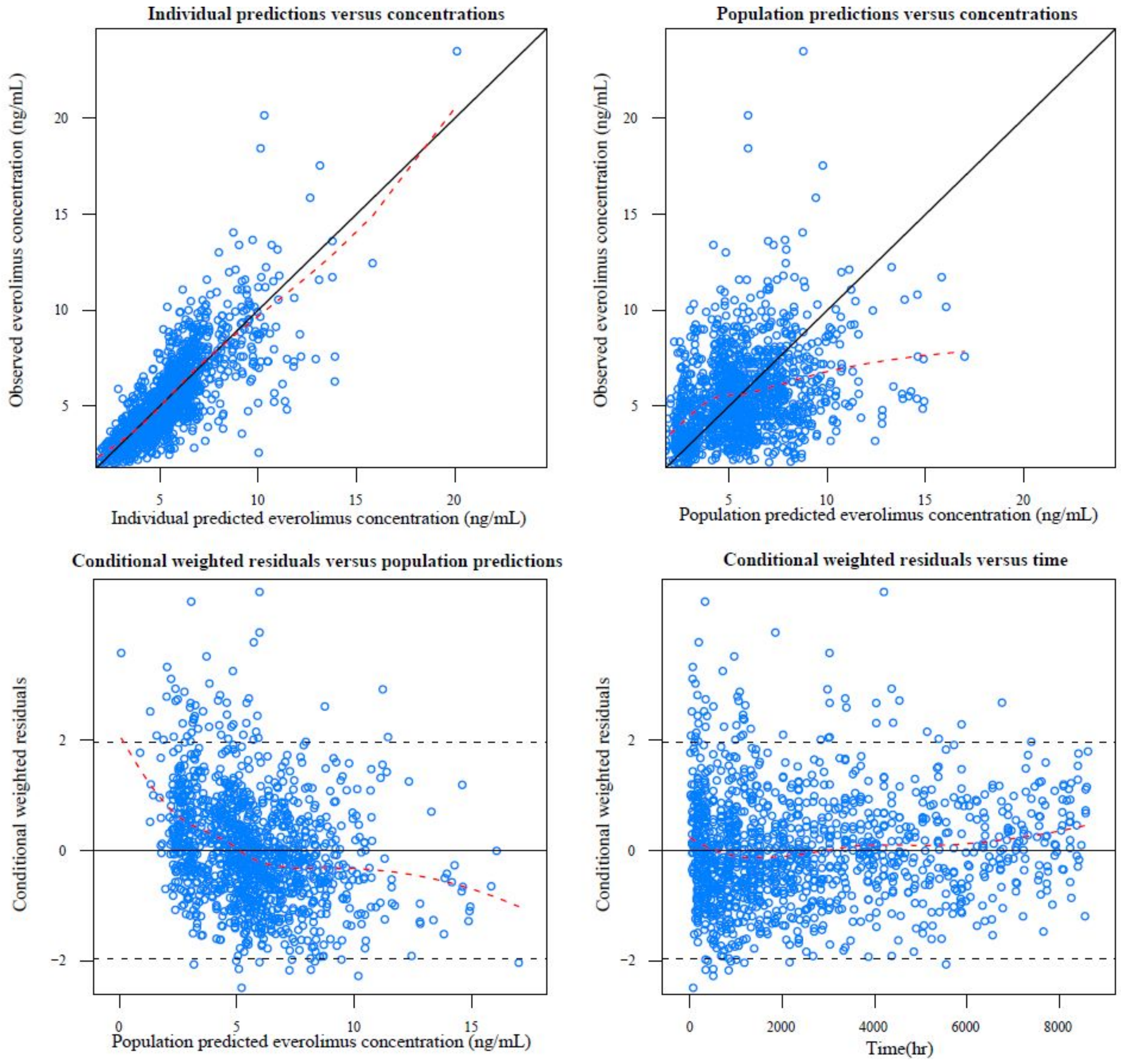

Supplement: Supplementary file 1 — pt4c00581_si_001.pdf [file pt4c00581_si_001.pdf]
